# Travel From Native Lands to US Abortion Facilities Before and After the *Dobbs v Jackson Women’s Health Organization* Decision

**DOI:** 10.1001/jamanetworkopen.2025.46883

**Published:** 2025-12-04

**Authors:** Rebecca Hailu Astatke, Lauren van Schilfgaarde, Miriam Jorgensen, Laura E. Dodge

**Affiliations:** 1 Department of Epidemiology, Harvard T.H. Chan School of Public Health, Boston, Massachusetts; 2 Department of Obstetrics and Gynecology, Beth Israel Deaconess Medical Center, Harvard Medical School, Boston, Massachusetts; 3 University of California, Los Angeles School of Law, University of California, Los Angeles; 4 Udall Center for Studies in Public Policy, Native Nations Institute at the University of Arizona, Tucson; 5 Kennedy School of Government, Harvard Project on Indigenous Governance and Development, Cambridge, Massachusetts

## Abstract

**Importance:**

Limited evidence exists concerning access to abortion facilities from Native lands. To address this gap, travel times from Native lands to abortion facilities before and after the *Dobbs v Jackson Women’s Health Organization* (*Dobbs*) decision were compared.

**Objective:**

To compare travel times to abortion facilities from Native lands before and after the *Dobbs* decision.

**Design, Setting, and Participants:**

This cross-sectional study, conducted from July 1, 2023, to September 30, 2025, measured the proportion of Native lands served by abortion facilities (coverage) and the minimum travel times to abortion facilities. For coverage, the census-designated Native lands (N = 650) within and outside 30-, 60-, and 90-minute drive times to an abortion facility were quantified. For minimum travel, the drive times from Tribal headquarters (N = 577) and Native land population-weighted centroids (N = 650) to the nearest facility were estimated.

**Main Outcomes and Measures:**

The primary outcomes were the estimates of the median travel time from Native lands to the nearest abortion facility and the pre- and post-*Dobbs* differences in the contiguous US. Primary analyses compared median drive times in the contiguous US before and after *Dobbs*. The pre-*Dobbs* period included facilities active in 2021, while the post-*Dobbs* period excluded facilities in states with total abortion bans (n = 14) or total or 6-week bans (n = 16) in effect as of October 31, 2023. For Alaska and Hawaii, Euclidean distance was estimated to account for varied travel modes; because no such bans were imposed, pre- and post-*Dobbs* differences were not examined.

**Results:**

Across 650 Native lands, with an estimated 950 991 female residents of reproductive age (15-44 years), less than half (289 [44.5%]) were within a 90-minute drive of any abortion facility after *Dobbs*. In the contiguous US, the median drive time significantly increased from 65.5 minutes (IQR, 31.9-103.1 minutes) before *Dobbs* to 72.3 minutes (IQR, 32.5-147.3 minutes) (total bans only) and 74.0 minutes (IQR, 32.6-152.4 minutes) (total and 6-week bans) after *Dobbs*. The median distance was 470.1 km (IQR, 270.7-665.9 km) in Alaska and 63.1 km (IQR, 22.9-179.1 km) in Hawaii, where 56.4% of Native lands (31 of 55) required travel to a different island.

**Conclusions and Relevance:**

This cross-sectional study of access to abortion facilities from Native lands found that individuals on Native lands faced disproportionately long travel times to abortion facilities, which became longer after *Dobbs.* Addressing these inequities is critical for advancing health equity for Indigenous communities.

## Key Points

**Question:**

How did geographic access from Native lands to abortion facilities change after the *Dobbs v Jackson Women’s Health Organization* (*Dobbs*) decision?

**Findings:**

In this cross-sectional study of 650 Native lands, with an estimated 950 991 female residents of reproductive age, nearly half of Native lands were over 90 minutes from an abortion facility before *Dobbs*, and the proportion increased after the decision; in the contiguous US, the minimum drive time to the nearest facility also increased significantly after *Dobbs*. In Alaska and Hawaii, access remained especially limited, with most Native lands requiring long-distance or interisland travel.

**Meaning:**

This study suggests that the *Dobbs* decision was associated with significantly longer minimum drive times to an abortion facility from Native lands.

## Teaser

This cross-sectional study compares travel times to abortion facilities from Native lands before and after the *Dobbs v Jackson Women’s Health Organization* decision.

## Introduction

Since 1976, the Hyde Amendment has effectively denied abortion care*—*an essential health service*—*to individuals who rely on, or are entitled to, federally funded health programs. This restriction includes those who receive care through the Indian Health Service, the principal source of co-located, comprehensive health care for American Indian and Alaska Native individuals living on Native lands (see eTable 1 in Supplement 1 for terminology notes).^1,2,3,4,5^ *Native lands* refers both to areas legally defined as “Indian country” and to other lands with substantial Native populations, such as those in Alaska and Hawaii. Outside Indian country, state laws have already resulted in disparate availability of abortion services for Native people.^1,2,3,6,7,8^ On June 24, 2022, *Dobbs v Jackson Women’s Health Organization* (hereafter, *Dobbs*), overturned *Roe v Wade*, eliminating the federal constitutional right to abortion, shifting regulatory authority to individual states and leading to widespread restrictions.^1^ Within the same week, *Oklahoma v Castro-Huerta* expanded state criminal authority over non-Indian populations into Indian country, exposing those who provide or facilitate abortion care in Indian country to state criminalization regardless of Tribal policy.^1,9^ Together, these decisions not only further limited abortion access for Native people but also undermine Tribal sovereignty, deepening inequities in reproductive health and autonomy.

Amid increased legal restrictions on abortion, travel requirements have become an even more critical factor in access to health care; increased travel times and distances are correlated with long delays and the inability to obtain abortion care.^10^ These challenges are magnified after *Dobbs*, with an increased percentage of females of reproductive age (15-44 years) living in census tracts more than 60 minutes from the nearest abortion facility compared with before *Dobbs*.^11^ Although all females living in the US experienced this change, the Native population stands out. Compared with other groups, a larger proportion of American Indian and Alaska Native individuals lived in census tracts more than 60 minutes away from abortion care.^11^ The data also show that census tract–based drive times increased by 20.4 percentage points for American Indian and Alaska Native females and by 11.8 percentage points for Native Hawaiian females.^11^

Although many American Indian and Alaska Native individuals live in urban areas, 2010 US Census data show that 68% of the single-race American Indian and Alaska Native population (ie, the population considered in the census tract–based spatial studies) lived on Native lands (42%) or surrounding counties (26%),^12^ emphasizing the importance of these distinct geographies in abortion access. US Census tract boundaries do not always correspond to Native land boundaries. Therefore, for residents of Native lands, a Native lands–based spatial analysis, as opposed to a census tract–based spatial analysis, is an alternative and potentially more accurate approach to estimating travel to abortion care. Most spatial analyses, moreover, limit their geographic scope to the contiguous US, excluding over one-third of Native lands. Accordingly, the present study seeks to describe travel to abortion facilities from Native lands in the US before and after the *Dobb*s decision.

## Methods

This is a secondary analysis of nonhuman and nonidentifiable public data; the institutional review board at Beth Israel Deaconess Medical Center determined this study to be exempt. We followed the Strengthening the Reporting of Observational Studies in Epidemiology (STROBE) reporting guideline for cross-sectional studies.

### Reflexivity

The study team consisted of 4 individuals, 1 trained in Tribal sovereignty and federal Indian law and legal scholarship, 1 trained in Native governance and economic development, and 2 trained in reproductive epidemiology. One author identifies as Native American, and the other 3 identify as settler scholars, 1 as Black and 2 as non-Hispanic White. All authors identify as female.

### Data Sources

We accessed the US Census Bureau’s 2020 Native lands geographic database and mapped a total of 650 legal and statistical census-designated Native lands. This mapping included the following 7 types of Native lands as defined by the US Census Bureau and Bureau of Indian Affairs: (1) Federal American Indian Reservations (AIR) or Off-Reservation Trust Land; (2) Joint-Use Federal AIR and State AIR; (3) Alaska Native Village Statistical Area; (4) Hawaiian Home Lands; (5) Oklahoma Tribal Statistical Area (OTSA); (6) State Designated Tribal Statistical Area; and (7) Joint-use OTSA. We obtained Tribal headquarters data (N = 577; 349 in the contiguous US and 228 in Alaska) from the National Tribal Geographic Information Systems Center and used these locations, which have been used in prior literature, to examine spatial access to other services among American Indian and Alaska Native communities as a proxy for residential locations on Native lands.^13,14^ To create a similar measure that was comparable across the contiguous US, Alaska, and Hawaii (given differences in modes of transportation outside the contiguous US and, in Hawaii, the lack of “headquarters” towns), we constructed population-weighted centroids (N = 650) as a proxy for where Native people reside. To construct centroids, we used the total US Census population, as our software did not allow us to combine the single-race American Indian and Alaska Native and Native Hawaiian or Other Pacific Islander populations with the relevant 2 or more races population. We chose total population rather than restricting to females of reproductive age to provide a more robust measure across varied Native lands. Some Tribal lands have very small populations (and thus large margins of errors in American Community Survey data), so limiting to females of reproductive age may less accurately estimates effects than using the total population. In addition to statistical considerations, abortion policy extends beyond individuals of reproductive age, affecting families and communities, including parents, children, and partners^15^; this approach also reflects an inclusive perspective that aligns with many Indigenous peoples’ holistic understandings of family, health, and well-being.

We obtained locations of abortion facilities from the Advancing New Standards in Reproductive Health database.^16^ With the *Dobbs* decision issued in June 2022, we defined the pre-*Dobbs* period as January to December 2021 and the post-*Dobbs* period as November 2023, approximately 18 months before and after the decision. Active facilities in 2021 were included in the pre-*Dobbs* period (N = 749). For the post-*Dobbs* period, we excluded facilities in states with abortion bans as of October 31, 2023, based on AbortionFinder’s state-by-state guide.^17^ This yielded 690 facilities after removing the 14 states with total bans (Alabama, Arkansas, Idaho, Indiana, Kentucky, Louisiana, Mississippi, Missouri, North Dakota, Oklahoma, South Dakota, Tennessee, Texas, and West Virginia), and 673 facilities after also removing those in the 2 states with 6-week bans (Georgia and South Carolina).^17^

### Study Design

To assess travel from Native lands to abortion care, we conducted a cross-sectional geospatial analysis from July 1, 2023, to September 30, 2025, applying 2 common accessibility measures: coverage and minimum travel.^18^ We measured the proportion of Native lands served by abortion facilities (coverage) and quantified travel burden (minimum travel) using a person-centered rather than clinician-centered approach.^19^

### Statistical Analysis

#### Coverage

First, we conducted a network analysis to generate areas that were a 30-, 60-, and 90-minute drive time around all abortion facilities. We used 60 minutes as the primary drive time threshold, consistent with prior literature and government standards for access to specialty care,^10,11,20,21^ and included 30- and 90-minute thresholds in sensitivity analyses to assess robustness. These areas were intersected with Native land areas to determine the proportion of Native lands with and without coverage before and after *Dobbs*. This approach was previously used by Juraska et al^20^ to assess reproductive health–related services coverage of Native lands inclusive of those in Alaska and Hawaii.

#### Minimum Travel

Second, we estimated travel from Tribal headquarters and population-weighted centroids to the nearest abortion facility. In the contiguous US, we estimated drive times before and after *Dobbs.* We found evidence against normality of the distribution of untransformed and log-transformed drive times using density plots, quantile-quantile plots, and the Shapiro-Wilk test. Therefore, we used the paired sample Wilcoxon signed rank test with a continuity correction to compare the pre- and post-*Dobbs* differences in median (IQR) drive times.

For Alaska and Hawaii, we estimated the straight-line (Euclidean) distance, as travel in these states often requires air transport and both states have a small number of abortion facilities. In addition, because neither state had a total ban or a 6-week ban enacted after *Dobbs*, we did not examine the pre- and post-*Dobbs* differences in minimum travel outside the contiguous US (accessibility measures are summarized in Table 1). We conducted 1-sided Wilcoxon signed rank tests, setting the significance level at α = .05. Analyses were conducted using R, version 4.5.1 (R Project for Statistical Computing), ArcGIS Pro, version 3.2 (Esri), and ArcGIS Online (Esri).

**Table 1.** Accessibility Measures Used to Assess Native Land Abortion Access

Measure and description	Distance calculation	Geographic scope	Origin (Native land geography)	Destination
Coverage				
Native lands within a 30-, 60-, and 90-min drive to an abortion facility before and after *Dobbs*	Network	Contiguous US, Alaska, and Hawaii	Any part of the Native land area that is 30, 60, or 90 min away	Any facility
Minimum travel				
Drive time to the nearest abortion facility before and after *Dobbs*, min	Network	Contiguous US	Tribal headquarters; population-weighted centroids^a^	Nearest facility
Distance to the nearest abortion facility, km^b^	Straight line (Euclidean)	Alaska and Hawaii^c^	Tribal headquarters; population-weighted centroids^a^	Nearest facility

Abbreviation: *Dobbs*, *Dobbs v Jackson Women’s Health Organization*.

^a^ Population-weighted centroids were constructed using the US 2020 Census total population on Native lands.

^b^ Construction of the sample of abortion facilities and calculation of Eucledian distance are described in the Methods section.

^c^ As the National Tribal Geographic Information Support Center data on Tribal headquarters does not include Hawaiian Home Lands, minimum travel analyses in Hawaii used only population-weighted centroids as the origin.

#### Outcome

The primary outcomes were the estimates of the median travel time from Native lands to the nearest abortion facility and the change before and after the *Dobbs* decision among lands in the contiguous US.

## Results

### Coverage

**Figure 1.**
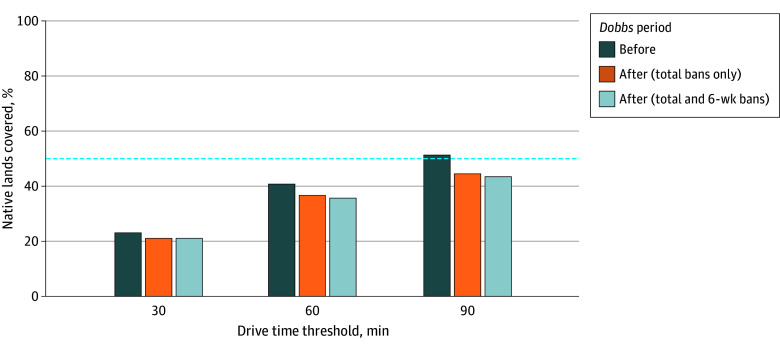
Coverage of Native Lands by Abortion Facilities Before and After the *Dobbs v Jackson Women’s Health Organization* Decision Percentage of Native lands with coverage before and after *Dobbs v Jackson Women’s Health Organization* decision (*Dobbs*), estimated from a repeated cross-sectional spatial analysis. Coverage was defined by whether any part of a Native land was within a 30-, 60-, or 90-minute drive time threshold of any abortion facility; such lands were considered covered, whereas those beyond this threshold were not. The dashed line indicates 50% of Native lands were covered.

**Figure 2.**
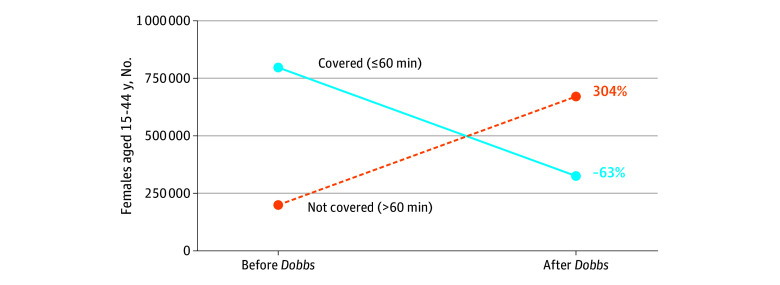
Sixty-Minute Coverage of Native Lands Population by Abortion Facilities After the *Dobbs v Jackson Women’s Health Organization* Decision Population counts and percentage change of US females of reproductive age (15-44 years) living on Native lands with and without coverage before and after the *Dobbs v Jackson Women’s Health Organization* decision (*Dobbs)*, estimated from a repeated cross-sectional spatial analysis. Among females living on Native lands, the number with coverage decreased from 788 370 of 950 991 (82.9%) in the pre-*Dobbs* period to 294 677 of 950 991 (31.0%) in the post-*Dobbs* period—a 62.6% decrease. Over the same period, the number without coverage increased from 162 621 of 950 991 (17.1%) to 656 314 of 950 991 (69.0%), a 303.6% increase. This represents an absolute decrease of 493 693 females with coverage, corresponding to a 51.9 percentage point decrease.

Across the 650 Native lands, the estimated total population was 5 114 393 individuals, of whom 950 991 were females of reproductive age. Coverage was limited both before and after *Dobbs*, with about half of Native lands within 90 minutes of an abortion facility. After *Dobbs*, the proportion of Native lands with coverage decreased modestly, from 23.2% (151 of 650) to 21.2% (138 of 650) within 30 minutes, from 40.8% (265 of 650) to 36.8% (293 of 650) within 60 minutes, and from 51.4% (334 of 650) to 44.5% (289 of 650) within 90 minutes (Figure 1; Table 2). However, changes among females of reproductive age were substantial. Within 60 minutes, the population with coverage decreased by 62.6% (from 788 370 to 294 677), while the population without coverage increased by 303.6% (from 162 621 to 656 314) (Figure 2; Table 2). Similar patterns were observed within 30 minutes (covered population decreased by 62.2% [from 629 840 to 238 375]; population without coverage increased by 121.9% [from 321 151 to 712 616]) and 90 minutes (covered population decreased by 61.3% [from 888 332 to 343 742]; population without coverage increased by 869.1% [from 62 659 to 607 249]), reflecting coverage loss among a few highly populated Native lands. Incorporating 6-week bans reduced coverage only slightly further across thresholds.

**Table 2.** Coverage of Native Lands Within 30-, 60-, or 90-Minute Drive Times of Any Abortion Facility and Demographic Characteristics Before and After the *Dobbs v Jackson Women’s Health Organization* Decision^a^^,^^b^

Characteristic	Coverage measures, No. (%)					
	January-December 2021 (pre-*Dobbs*)		November 2023 (post-*Dobbs* total bans only)		November 2023 (post-*Dobbs* total and 6-wk bans)	
	>Threshold	≤Threshold	>Threshold	≤Threshold	>Threshold	≤Threshold
**30-min Coverage threshold**						
Native lands (N = 650)	499 (76.8)	151 (23.2)	512 (78.8)	138 (21.2)	513 (78.9)	137 (21.1)
Land area, mean (SD), km^2^	579.2 (2357.7)	1430.6 (6018.3)	729.1 (2804.0)	954.0 (5582.3)	727.8 (2799.9)	961.0 (5603.8)
Total population (N=5 114 393)^c^	1 751 886 (34.3)	3 362 507 (65.7)	3 851 905 (75.3)	1 262 488 (24.7)	3 858 174 (75.4)	1 256 219 (24.6)
Females aged 15-44 y (n = 950 991)^c^	321 151 (33.8)	629 840 (66.2)	712 616 (74.9)	238 375 (25.1)	714 020 (75.1)	236 971 (24.9)
**60-min Coverage threshold**						
Native lands (N = 650)	385 (59.2)	265 (40.8)	411 (63.2)	239 (36.8)	417 (62.2)	233 (35.8)
Land area, mean (SD), km^2^	522.5 (2193.7)	1146.9 (4913.5)	765.3 (2863.9)	797.5 (4545.5)	755.1 (2845.4)	815.0 (4604.4)
Total population (N = 5 114 393)^c^	896 816 (17.5)	4 217 577 (82.5)	3 539 560 (69.2)	1 574 833 (30.8)	3 565 958 (69.7)	1 548 435 (30.3)
Females aged 15-44 y (n = 950 991)^c^	162 621 (17.1)	788 370 (82.9)	656 314 (69.0)	294 677 (31.0)	661 539 (69.6)	289 452 (30.4)
**90-min Coverage threshold**						
Native lands (N = 650)	316 (48.6)	334 (51.4)	361 (55.5)	289 (44.5)	367 (56.5)	283 (43.5)
Land area, mean (SD), km^2^	356.2 (1412.1)	1175.4 (4759.2)	714.2 (2848.3)	854.6 (4312.7)	1132.1 (4550.4)	872.4 (4357.5)
Total population (N = 5 114 393)^c^	335 008 (6.6)	4 779 385 (93.4)	3 266 496 (63.9)	1 847 897 (36.1)	3 285 101 (64.2)	1 829 292 (35.8)
Females aged 15-44 y (n = 950 991)^c^	62 659 (6.6)	888 332 (93.4)	607 249 (63.9)	343 742 (36.1)	610 849 (64.2)	340 142 (35.8)

Abbreviation: *Dobbs*, *Dobbs v Jackson Women’s Health Organization*.

^a^ Coverage was defined by whether any part of a Native land was within a 30-, 60-, or 90-minute drive of any abortion facility; such lands were considered covered, whereas those beyond this threshold were not.

^b^ The pre-*Dobbs* period (January-December 2021) included all active abortion facilities in 2021. The post-*Dobbs* period (beginning November 2023) was constructed by removing facilities in states with total bans only (n = 14) or total or 6-week abortion bans (n = 16) as of October 31, 2023.

^c^ Total population and females of reproductive age (15-44 years) counts were from the US 2020 Census.

### Minimum Travel

In the contiguous US, median drive times significantly increased after *Dobbs*: from 57.4 minutes (IQR, 28.4-102.6 minutes) to 66.6 minutes (IQR, 29.0-143.8 minutes) from Tribal headquarters and from 65.5 minutes (IQR, 31.9-103.1 minutes) to 72.3 minutes (IQR, 32.5-147.3 minutes) from population-weighted centroids (Table 3; eFigure in Supplement 1). Under the post-*Dobbs* scenario incorporating total and 6-week bans, median drive times from Tribal headquarters remained unchanged, while median drive times from population-weighted centroids increased further to 74.0 minutes (IQR, 32.6-152.4 minutes).

**Table 3.** Minimum Travel From Native Lands to Abortion Facilities Before and After *Dobbs v Jackson Women’s Health Organization* Decision^a^

Geographic scope	January-December 2021 (pre-*Dobbs*)	November 2023 (post-*Dobbs *total bans only)	November 2023 (post-*Dobbs *total and 6-wk bans)
Contiguous US, median (IQR), min			
Tribal headquarters	57.4 (28.4-102.6)	66.6 (29.0-143.8)	66.6 (29.0-143.8)
Population-weighted centroids	65.5 (31.9-103.1)	72.3 (32.5-147.3)	74.0 (32.6-152.4)
Alaska, median (IQR), km^b^			
Tribal headquarters	472.5 (265.1-684.8)	NA	NA
Population-weighted centroids	470.1 (270.7-665.9)	NA	NA
Hawaii, median (IQR), km^b^			
Population-weighted centroids^c^	63.1 (22.9-179.1)	NA	NA

Abbreviations: *Dobbs*, *Dobbs v Jackson Women’s Health Organization*; NA, not applicable.

^a^ In the contiguous US, drive times (minutes) were estimated for minimum travel using network distance calculations. In Alaska and Hawaii, where different modes of travel other than driving are likely used, distance (kilometers) were estimated for minimum travel using straight-line (Euclidean) distance calculations.

^b^ As neither Alaska nor Hawaii enacted total or 6-week abortion bans, pre- and post-*Dobbs* differences were not examined.

^c^ As the National Tribal Geographic Information Support Center data on Tribal headquarters does not include Hawaiian Home Lands, minimum travel analyses in Hawaii used only population-weighted centroids as the origin.

Outside the contiguous US, there were a small number of abortion facilities. Alaska had 5 located centrally, and Hawaii had 3 facilities across 2 of the 7 inhabited islands as of 2021.

In Alaska, only 5 of 207 Native lands (2.4%)—those near Anchorage, Fairbanks, Juneau, and Soldotna—were within 30 minutes of an abortion facility, and just 8 of 207 (3.9%) were within 60 or 90 minutes. The median distance to the nearest facility was more than 466.7 km from Tribal headquarters (472.5 km [IQR, 265.1-684.8 km]) and population-weighted centroids (470.1 km [IQR, 270.7-665.9 km]) (Table 3).

In Hawaii, all 24 Native lands on Oahu and Maui were within 90 minutes of an abortion facility; 22 (91.7%) were within 60 minutes, and 5 (20.8%) were within 30 minutes. The median distance to the nearest abortion facility from population-weighted centroids was 63.1 km (IQR, 22.9-179.1 km) (Table 3). Although distances are shorter, many Hawaiian Home Lands (56.4% [31 of 55]) are on islands without an abortion facility, requiring interisland travel to access care (eTable 2 in Supplement 1).^22^

## Discussion

This study characterized geographic abortion access from Native lands and examined how access changed after the *Dobbs* decision. We found that abortion facilities do not adequately serve Native lands, with most located beyond the recommended 60-minute travel standard.

Even prior to *Dobbs*, nearly half of Native lands were more than a 90-minute drive from the nearest abortion facility, underscoring longstanding structural barriers to timely abortion care. After *Dobbs*, the proportion of Native lands with coverage decreased modestly across 30-, 60-, and 90-minute coverage thresholds. However, the number of people affected increased sharply, with the population of females of reproductive age without 60-minute coverage increasing by more than 300%.

Regarding Native lands within the contiguous US, the *Dobbs* decision was associated with a longer travel time to the nearest abortion facility. The median estimated drive time increased from 65.5 minutes before *Dobbs* to 72.3 minutes after *Dobbs* when accounting for total bans only and to 74.0 minutes when incorporating total and 6-week bans. By comparison, one study of all census tracts in the contiguous US reported much shorter median travel times—10.9 minutes before *Dobbs* and 17.0 minutes after *Dobbs* (total and 6-week bans)—underscoring that individuals on Native lands face disproportionately greater travel burdens before and after *Dobbs*.^11^

Outside the contiguous US, abortion access was even more limited. In Alaska, the median Euclidean distance to the nearest facility exceeded 466 km, and less than 5% of Native lands were within a 90-minute drive to an abortion facility. In Hawaii, abortion facilities were accessible by vehicle for the 24 lands on Oahu and Maui, yet only 5 (20.8%) were within 30 minutes of a facility. The remaining lands were on islands without an abortion facility, necessitating interisland travel to obtain care.

These geographic barriers for Native communities, especially those in Alaska and Hawaii, underscore the need for research on patients’ perspectives and experiences with reproductive health care—such as how they navigate multimodal travel, where they currently seek care, and their preferences for abortion services as part of comprehensive health care—to better address the abortion access challenges facing Native individuals across the US.

### Limitations

This study has several important limitations. First, we characterized spatial accessibility in the contiguous US by vehicle. A spatial approach to abortion access is necessary but partial.^19^ Although outside the scope of this study, we did not account for other pertinent factors associated with individuals’ access to abortion services, such as financial resources, alternatives in service delivery (including receipt of medication via mail), and other modes of travel, which are particularly relevant in Alaska, Hawaii, certain rural areas, restrictive policy contexts, or other parts of the continental US. In addition, our use of street network data from Esri may not fully capture Tribal roads.^13^ Second, this study does not include data on the Native population on Native lands. In the absence of data on Native individuals’ location or health services access, we used Tribal headquarters and population-weighted centroids of the total population on Native lands. Third, we did not capture abortion access for those who do not live on Native lands. Although we recognize the importance of abortion access for all Indigenous people residing in the US, the foregoing studies describe the situation for different racial and ethnic groups in the US as a whole, but the disproportionately more challenging situation of the Native lands’ population had not yet been described. Fourth, in considering multiple lenses of Indigenous political and racial identity, we may be too inclusive or exclusive at times. In particular, US Census data are limited in capturing Indigenous people in that they consider only racial identity and not legal or political identity (ie, the data do not account for Tribal nation citizenship, which is uniquely defined by each Tribal nation). US Census data also undercount Indigenous people, especially those in rural areas or on and near Native lands.^23,24,25^ The US Census nonetheless offers the most complete count of the population on Native lands.^13^ Fifth, the landscape of abortion access is changing rapidly as states pass additional legislation to expand or restrict abortion access in the wake of the *Dobbs* decision; thus, the locations included in the present study may not accurately reflect facilities available at later dates.

## Conclusions

In this cross-sectional study of access to abortion facilities from Native lands, we found that travel times from Native lands to the nearest abortion facility were substantial. Almost half of Native lands were more than a 90-minute drive to any abortion facility before *Dobbs*. After *Dobbs,* coverage decreased and minimum travel increased significantly. Our results provide empirical evidence of the spatial inequities in abortion access from Native lands and the consequential outcome of repealing *Roe v Wade*.

## Notes

Supplement 1. **eTable 1.** Glossary of Key Terms **eFigure.** Minimum Drive Time From Native Lands in the Contiguous US to an Abortion Facility Before and After the *Dobbs v Jackson Women's Health* Decision **eTable 2.** Hawaiian Home Lands and Abortion Facilities by Island, 2021

Supplement 2.
Data Sharing Statement
