# Supplementary material for: Travel From Native Lands to US Abortion Facilities Before and After the Dobbs v Jackson Women’s Health Organization Decision
Source: JAMA Netw Open. 2025 Dec 4;8(12):e2546883. doi: 10.1001/jamanetworkopen.2025.46883 (PMC12679323; doi:10.1001/jamanetworkopen.2025.46883)
Supplement: Supplement 1. — eTable 1. Glossary of Key Terms eFigure. Minimum Drive Time From Native Lands in the Contiguous US to an Abortion Facility Before and After the Dobbs v Jackson Women's Health Decision eTable 2. Hawaiian Home Lands and Abortion Facilities by Island, 2021 [file jamanetwopen-e2546883-s001.pdf]

Supplementary Online Content

Astatke RH, van Schilfgaarde L, Jorgensen M, Dodge LE. Travel from Native lands to US abortion facilities before and after the *Dobbs v Jackson Women's Health Organization* decision. *JAMA Netw Open.* 2025;8(12):e2546883. doi:10.1001/jamanetworkopen.2025.46883

eTable 1. Glossary of Key Terms
eFigure. Minimum Drive Time From Native Lands in the Contiguous US to an Abortion Facility Before and After the *Dobbs v Jackson Women's Health* Decision
eTable 2. Hawaiian Home Lands and Abortion Facilities by Island, 2021

This supplementary material has been provided by the authors to give readers additional information about their work.

© 2025 Astatke RH et al. *JAMA Network Open.*

**eTable 1.** Glossary of Key Terms	
Term	Description and context
Indigenous peoples and lands	This Article uses multiple terms when referring to the Indigenous people of the United States, including American Indian and Alaska Native, Native Hawaiian and Pacific Islander, Native, Indian, and Indigenous. Each of these can be appropriate depending on the context. This Article capitalizes these terms, as well as the terms "Tribe" and "Tribal." Within the US, members of federally recognized Tribes are both a racial class and a political class, which triggers federal obligations, including the provision of health care. Additionally, this Article discusses multiple forms of identity (political, legal, and racial) for Indigenous peoples. We refer to the various Native lands using numerous terms as well. These terms include federally recognized, non-federally recognized, legal, and statistical Native lands. The term "Native lands" is intended to capture lands legally defined as "Indian country," as well as lands which form the statistically-relevant concentrated homelands of Indigenous communities but which are not defined as "Indian country," such as in Alaska and Hawaii. We have cited the First Nations Development Institute, which uses population numbers for American Indian and Alaska Native alone and not in combination with any other race and Rader et al., which examines American Indian and Alaska Native alone, Hawaiian or Pacific Islander alone, and the two or more races populations.[11,12] Notably, the single race ("alone") category tracks the multi-race population data for American Indians and Alaska Natives more closely in rural and reservation areas than in non-rural and non-reservation areas. Nonetheless, because Indigenous people in the United States identify as two or more races at significantly higher rates than other racial and ethnic groups, scholars increasingly recommend using the "alone" and "two or more races" data in combination for an accurate and equitable enumeration of the US Indigenous population.[4] These inclusions and exclusions have important implications for data comparability. The census generally undercounts the US population, as it relies on self-reporting, with noted undercounts of Indigenous people, those residing in rural areas, and those residing on Native lands.[23-25] While we are aware of these enumeration issues and want readers to be aware of them, data analyses reported in this paper rely on counts of the total population (all races) living on Native lands because all Native land residents—Indigenous and non-Indigenous—are subject to the access problems described herein. Furthermore, this Article recognizes that not all Indigenous people seeking abortion care are women. This Article uses gender-inclusive language when referring to individuals but not when quoting or describing the substance of a source. For example, we refer to individuals of reproductive age as females aged 15–44 years when referencing US Census data used.
Oklahoma v. Castro-Huerta	*Oklahoma v. Castro-Huerta,* 597 US 629 (2022) issued within a week of the *Dobbs* decision, recognized state criminal jurisdiction over crimes against Indian people committed in Indian country (as defined under federal law) by non-Indian perpetrators, consequently infringing on Tribal self-determination and sovereignty.[1] Before *Castro-Huerta,* only Tribes and the federal government possessed criminal jurisdiction to prosecute crimes committed against Native persons, unless the state otherwise had recognized jurisdiction in a statute, such as states in which Public Law 280 applied. As a result, if a state were to criminalize abortion care, such a criminal prohibition could be extended to non-Indians throughout Indian country, likely impacting non-Indian clinicians.[1]
Dobbs v. Jackson Women's Health Organization	*Dobbs v. Jackson Women's Health Organization,* 597 US 215 (2022), overruled both the 1973 *Roe v. Wade* and 1992 *Planned Parenthood v. Casey* decisions, to hold that the US Constitution does not confer a substantive right to abortion and instead gives individual states the full power to regulate any aspect of abortion not protected by federal law.[1]

© 2025 Astatke RH et al. *JAMA Network Open.*

eFigure. Minimum Drive Time From Native Lands in the Contiguous US to an Abortion Facility Before and After the *Dobbs v Jackson Women's Health* Decision

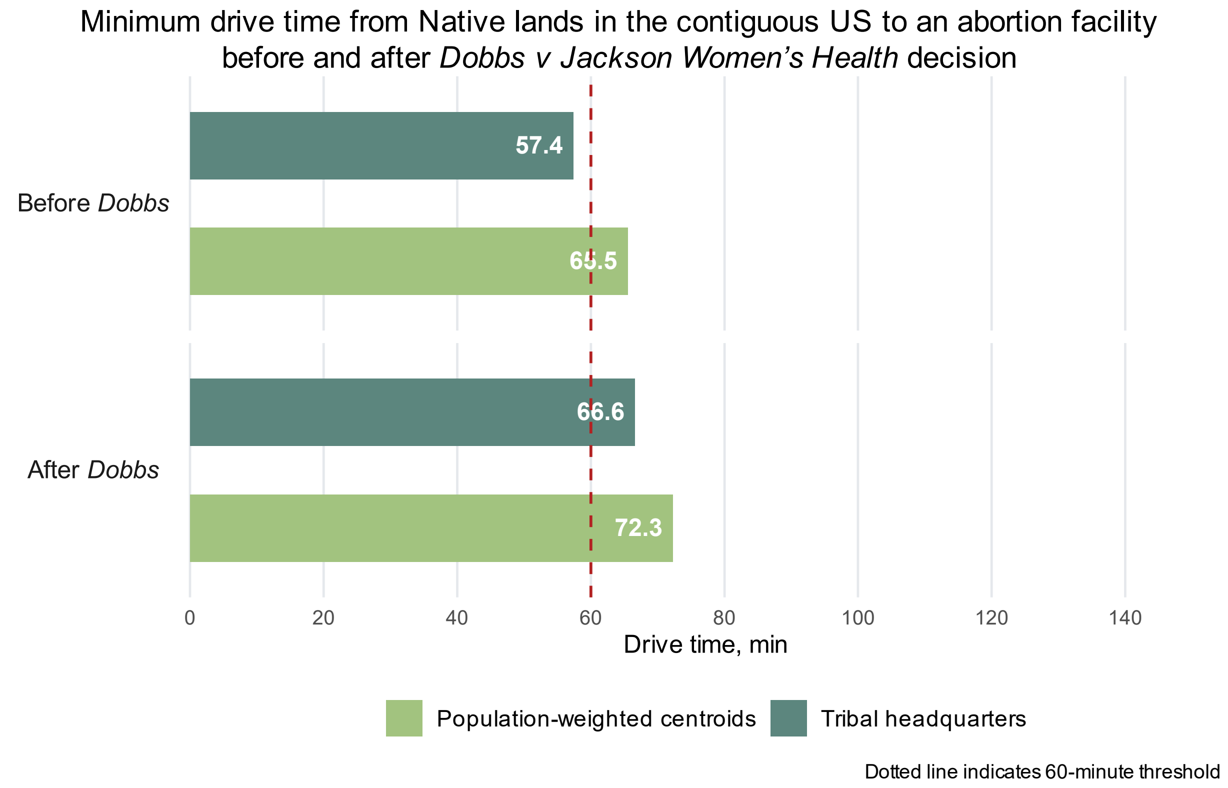

eFigure. Legend.
Median (interquartile range, IQR) drive time from Tribal headquarters and Native land population-weighted centroids to the nearest abortion facility before and after *Dobbs*, estimated from a repeated cross-sectional spatial analysis. The pre-*Dobbs* period (January-December 2021) included all active abortion facilities in 2021. The post-*Dobbs* period (November 2023) was constructed by removing facilities in states with total bans only (n=14) or total or 6-week abortion bans (n=16) as of October 31, 2023.

© 2025 Astatke RH et al. *JAMA Network Open.*

**eTable 2.** Hawaiian Home Lands and Abortion Facilities by Island, 2021		
**Island**	**Hawaiian Home Lands, No. (%)**	**Abortion facilities, No. (%)**
Oahu	17 (30.9)	2 (66.7)
Maui	7 (12.7)	1 (33.3)
Hawaii	18 (32.7)	0
Kauai	6 (10.9)	0
Molokai	6 (10.9)	0
Lanai	1 (1.8)	0

Counts include Hawaiian Home Lands and abortion facilities as of 2021on the six Hawaiian islands with designated Home Lands (excluding Niihau).
