# Supplementary material for: Travel From Native Lands to US Abortion Facilities Before and After the Dobbs v Jackson Women’s Health Organization Decision
Source: JAMA Netw Open. 2025 Dec 4;8(12):e2546883. doi: 10.1001/jamanetworkopen.2025.46883 (PMC12679323; doi:10.1001/jamanetworkopen.2025.46883)
Supplement: Supplement 2. — Data Sharing Statement [file jamanetwopen-e2546883-s002.pdf]

Data Sharing Statement

Astatke. Travel From Native Lands to US Abortion Facilities Before and After the Dobbs v Jackson Women's Health Organization Decision. *JAMA Netw Open*. Published December 04, 2025. doi:10.1001/jamanetworkopen.2025.46883

Data
Data available: No

Additional Information
Explanation for why data not available: Data on abortion facilities are proprietary to Advancing New Standards in Reproductive Health and cannot be shared. All other data used in this study are publicly available through their original sources.
